# Severe Combined Vitamin B12 and Folate Deficiency Presenting With Features Suggestive of Hemolytic Anemia in Two Long-Term Proton Pump Inhibitor Users: A Case Report

**DOI:** 10.7759/cureus.113398

**Published:** 2026-07-26

**Authors:** Anupom Kanti Paul, Nirupa Mestry

**Affiliations:** 1 General Internal Medicine, University Hospitals of Derby and Burton NHS Foundation Trust, Derby, GBR

**Keywords:** folate deficiency, hemolytic anemia, ineffective erythropoiesis, megaloblastic anemia, proton pump inhibitor, vitamine b12

## Abstract

Megaloblastic anemia may present with biochemical features suggestive of hemolysis, creating diagnostic uncertainty. We describe two middle-aged women who presented with severe symptomatic macrocytic anemia, markedly elevated lactate dehydrogenase (LDH), undetectable haptoglobin, and low reticulocyte counts. Further investigation confirmed a combined vitamin B12 and folate deficiency. No definitive underlying cause was identified, although long-term proton pump inhibitor (PPI) use was considered a potential contributing factor to vitamin B12 deficiency.

Both patients were treated with vitamin replacement therapy and discontinuation of PPI therapy, resulting in rapid clinical and hematological improvement. These cases highlight that combined vitamin B12 and folate deficiency may mimic hemolytic anemia and that the combination of elevated LDH, low haptoglobin, and a low reticulocyte count should raise suspicion of ineffective erythropoiesis rather than true hemolysis.

## Introduction

Vitamin B12 (cobalamin) absorption requires gastric acid and pepsin to release the vitamin from dietary proteins [1]. Long-term proton pump inhibitor (PPI) therapy increases gastric pH, which may impair this process and lead to clinically significant deficiency over time [2,3]. Folate absorption, by contrast, is largely independent of gastric acidity, as it primarily occurs in the proximal small intestine. Severe megaloblastic anemia can present with markedly elevated lactate dehydrogenase (LDH) and low haptoglobin levels due to ineffective erythropoiesis (intramedullary hemolysis), which may mimic microangiopathic or autoimmune hemolytic anemia [4]. In this process, defective erythroid precursors undergo premature destruction within the bone marrow, resulting in biochemical features suggestive of hemolysis despite an inappropriately low reticulocyte count [4]. Increased cellular turnover in severe cases may also increase folate demand, potentially exacerbating folate deficiency [5]. Although vitamin B12 deficiency presenting with features suggestive of hemolysis has been previously described, reports of severe combined vitamin B12 and folate deficiency in long-term PPI users remain limited. We report two such cases to highlight the diagnostic challenges and the importance of distinguishing ineffective erythropoiesis from true hemolysis.

## Case presentation

Patient 1

A 44-year-old Caucasian woman presented with a month-long history of progressive dyspnea, anxiety, and malaise. Her medical history included diabetes mellitus (diet-controlled) and gastro-esophageal reflux disease, the latter for which she had been taking omeprazole (a PPI) for six years. On examination, she was anemic but had no other significant systemic or neurological abnormalities.

Investigations revealed severe macrocytic anemia (hemoglobin 65 g/L; mean corpuscular volume (MCV) 115.1 fL), markedly elevated LDH (3509 U/L), and undetectable haptoglobin (<0.10 g/L), raising suspicion for hemolysis. The reticulocyte count was low (28.2 × 10⁹/L), suggesting ineffective erythropoiesis. Peripheral blood film demonstrated features consistent with megaloblastic anemia due to vitamin B12 and folate deficiency, including macrocytosis, hypersegmented neutrophils, and marked anisopoikilocytosis. Occasional nucleated red blood cells were observed, in keeping with ineffective erythropoiesis.

Further evaluation confirmed severe vitamin B12 deficiency (<100 ng/L) with low folate levels (1.0 ug/L). Autoimmune screening, including direct antiglobulin testing (DAT) and anti-intrinsic factor and parietal cell antibodies, was negative. Celiac serology was negative, and there was no history of gastrointestinal surgery, alcohol misuse, dietary restriction, or other recognized risk factors for nutritional deficiency. Thyroid function tests were normal, excluding hypothyroidism as a cause of macrocytic anemia. In the absence of an identified alternative cause, long-term PPI use was considered a likely contributing factor, although seronegative pernicious anemia could not be completely excluded. The key clinical characteristics and laboratory findings for both patients are summarized in Table 1.

**Table 1:** Summary data and investigations PPI: proton pump inhibitor; LDH: lactate dehydrogenase; TSH: thyroid-stimulating hormone

Data	Reference Range	Patient 1	Patient 2
Ethnicity		Caucasian	Caucasian
Duration of PPI Use		6 Years (Omeprazole)	9 years (Lansoprazole)
Autoantibodies (anti-intrinsic factor/parietal-cell antibodies)		Negative	Not tested
DAT (direct antiglobulin test)		Negative	Negative
Peak LDH (U/L)	0-250	3509	3622
Haptoglobin (g/L)	0.30-2.00	<0.10	<0.10
Reticulocytes (10*9/L)	50-100	28.2	18.5
TSH (mU/L)	0.3-5.5	1.53	0.47
Blood Iron (umol/L)	5.83-34.5	24	13
Ferritin (ug/L)	15-150	498	445
Transferrin (g/L)	2.0-3.2	Not recorded	2.25
Transferrin Saturation (%)	(20-40)%	Not recorded	25.7
Vitamin B12 (ng/L)	197-771	<100	116
Folate (ug/L)	3.8-26.8	1.0	1.0

The patient received two units of red blood cell transfusions because of disabling symptoms related to anemia and subsequently self-discharged. She was started on intramuscular vitamin B12 followed by oral folate supplementation with concurrent discontinuation of omeprazole. Marked hematological recovery was observed at the six-week follow-up, with serial hematological parameters shown in Table 2.

**Table 2:** Patient 1 hematological recovery Hb: hemoglobin; WBC: white blood cell count; PLT: platelet count; MCV: mean corpuscular volume; MCH: mean corpuscular hemoglobin; MCHC: mean corpuscular hemoglobin concentration; RBC: red blood cell count; HCT: hematocrit

Variable	Reference Range	Day 0 (Presentation)	Day 1 (Post 2 unit RBC transfusions )	Week 6 (Follow-up)
Hb (g/L)	120-150	65	86	129
WBC (10*9/L)	4.0-10.0	6.19	5.12	8.83
PLT (10*9/L)	150-410	174	150	349
MCV (fL)	83-101	115.1	105.5	97.4
MCH (pg)	27-32	39.2	36.1	30.6
MCHC (g/L)	315-345	340	343	314
RBC ( 10*12/L)	3.8-4.8	1.66	2.38	4.22
HCT(L/L)	0.360-0.460	0.191	0.251	0.411
Neutrophils (N) (10*9/L)	2.0-7.0	3.28	2.88	5.44
Lymphocytes (L) (10*9/L)	1.0-3.0	2.60	2.00	2.49
Monocytes (M) (10*9/L)	0.2-1.0	0.20	0.17	0.73
Eosinophils (E) ( 10*9/L)	0.02-0.50	0.11	0.06	0.14
Basophils (B) (10*9/L)	0.02-0.10	0.00	0.01	0.03

Patient 2

A 54-year-old Caucasian woman presented with a four-month history of fatigue, dyspnea, and a sensation of “stomach lumps.” Her history included chronic obstructive pulmonary disease, asthma, and gastroesophageal reflux disease, and she had been on long-term lansoprazole therapy for nine years for the latter. Physical examination revealed mild abdominal tenderness but no palpable masses or other abnormalities.

Laboratory investigations demonstrated severe macrocytic anemia (hemoglobin 71 g/L; MCV 102 fL), markedly elevated LDH (3622 U/L), and undetectable haptoglobin (<0.10 g/L), again suggestive of hemolysis. The reticulocyte count was low (18.5 × 10⁹/L), indicating ineffective erythropoiesis. Peripheral blood film demonstrated features consistent with megaloblastic anemia due to vitamin B12 and folate deficiency, including macrocytosis, hypersegmented neutrophils, and marked anisopoikilocytosis, along with thrombocytopenia and no evidence of red cell fragmentation.

Vitamin assays revealed vitamin B12 deficiency (116 ng/L) and concomitant folate deficiency (1.0 ug/L). Cross-sectional imaging with CT of the abdomen and pelvis to investigate the lump further did not identify any structural pathology. The celiac screen and fecal elastase were normal, excluding malabsorptive causes. The patient reported no dietary restrictions, alcohol misuse, or features suggestive of malnutrition. DAT was negative. Normal thyroid function tests made hypothyroidism an unlikely cause of the macrocytic anemia. However, the patient self-discharged before intrinsic factor and parietal cell antibody testing could be undertaken.

With no other identifiable etiologies, chronic PPI use was considered a likely contributing factor. However, pernicious anemia could not be excluded because intrinsic factor and parietal cell antibody testing were not performed. Lansoprazole was discontinued, and intramuscular vitamin B12 followed by oral folate replacement therapy commenced, resulting in significant clinical and hematological improvement, noticed at the four-week follow-up. The patient’s hematological recovery is summarized in Table 3.

**Table 3:** Patient 2 hematological recovery Hb: hemoglobin; WBC: white blood cell count; PLT: platelet count; MCV: mean corpuscular volume; MCH: mean corpuscular hemoglobin; MCHC: mean corpuscular hemoglobin concentration; RBC: red blood cell count; HCT: hematocrit

Variable	Reference Range	Day 0 (Presentation)	Week 4 (Follow-up)
Hb (g/L)	120-150	71	111
WBC ( 10*9/L)	4.0-10.0	3.39	5.58
PLT ( 10*9/L)	150-410	79	208
MCV (fL)	83-101	102	93
MCH (pg)	27-32	35.5	28.9
MCHC (g/L)	315-345	347	311
RBC (10*12/L)	3.8-4.8	2.0	3.84
HCT(L/L)	0.360-0.460	0.204	0.357
Neutrophils (N) ( 10*9/L)	2.0-7.0	2.31	3.12
Lymphocytes (L) (10*9/L)	1.0-3.0	0.91	1.86
Monocytes (M) (10*9/L)	0.2-1.0	0.09	0.36
Eosinophils (E) ( 10*9/L)	0.02-0.50	0.08	0.19
Basophils (B) ( 10*9/L)	0.02-0.10	0.00	0.05

## Discussion

Severe megaloblastic anemia can present with biochemical features that closely mimic hemolytic anemia, creating diagnostic uncertainty [4,6-8]. In our cases, markedly elevated LDH (>3500 U/L) and undetectable haptoglobin initially suggested hemolytic anemia. However, the low reticulocyte counts (<30 × 10⁹/L) indicated ineffective erythropoiesis rather than typical peripheral hemolysis [4]. This distinction is clinically important, as misinterpretation may lead to unnecessary and potentially invasive investigations for hemolytic disorders.

In vitamin B12 and folate deficiency, impaired DNA synthesis results in defective nuclear maturation of erythroid precursors, producing large, structurally abnormal megaloblasts [1,5]. These cells undergo premature destruction within the bone marrow, a process referred to as intramedullary hemolysis or ineffective erythropoiesis [4]. This mechanism leads to substantial release of intracellular enzymes such as LDH into the circulation [4]. The elevated ferritin levels observed in both patients may further support this mechanism, reflecting increased iron recycling from intramedullary destruction of erythroid precursors, although an acute-phase response may also have contributed. At the same time, the failure of erythroid maturation results in reduced output of functional red blood cells, reflected by an inappropriately low reticulocyte count. The combination of elevated LDH, low haptoglobin, and low reticulocyte response constitutes a possible hemolytic picture, which should be differentiated from typical peripheral hemolysis where reticulocytosis is expected [4,6,8].

Notably, Patient 2 demonstrated only modest macrocytosis (MCV 102 fL) despite profound combined vitamin B12 and folate deficiency. Although red blood cell distribution width (RDW) was not available, the peripheral blood film showed marked anisopoikilocytosis, suggesting a heterogeneous red-cell population that may have attenuated the degree of macrocytosis reflected by the MCV. In addition, the concurrent thrombocytopenia and mild leucopenia are consistent with megaloblastic pancytopenia and further support the diagnosis of severe megaloblastic anemia.

Several alternative diagnoses were considered. Autoimmune hemolytic anemia was considered because of the markedly elevated LDH and reduced haptoglobin; however, DAT was negative in both patients, and the reticulocyte response was inappropriately low, arguing against typical peripheral immune-mediated hemolysis. Thrombotic microangiopathy was also considered, particularly in Patient 2, because of the associated thrombocytopenia. However, the absence of red-cell fragmentation on the peripheral blood film, together with profound vitamin B12 and folate deficiency and a low reticulocyte count, favored ineffective erythropoiesis secondary to megaloblastic anemia. Myelodysplastic syndrome and other bone-marrow disorders may also produce macrocytosis and cytopenias, but the characteristic megaloblastic blood-film findings, documented vitamin deficiencies, and subsequent hematological recovery following vitamin replacement made these diagnoses less likely. Hypothyroidism was unlikely because thyroid function was normal in both patients.

Alternative causes of the vitamin deficiencies were also considered. Autoimmune gastritis remained a possibility, particularly because negative anti-intrinsic factor antibodies do not completely exclude the diagnosis. Patient 1 had negative intrinsic-factor and parietal-cell antibodies, whereas these tests were not obtained in Patient 2. Celiac disease was considered, but serology was negative; Patient 2 also had a normal fecal elastase. Neither patient reported gastrointestinal surgery, significant alcohol consumption, or dietary restriction. Thus, although several recognized causes were not supported by the available history and investigations, an unidentified nutritional or malabsorptive contribution cannot be completely excluded.

Chronic PPI use was considered a potential contributing factor to vitamin B12 deficiency in both patients. However, pernicious anemia could not be definitively excluded in Patient 2, while seronegative pernicious anemia remains a possibility in Patient 1. Gastric acid is essential for cleaving vitamin B12 from dietary protein, allowing it to bind to intrinsic factor and be absorbed in the terminal ileum [1]. Prolonged suppression of gastric acid secretion can impair this process, leading to gradual depletion of vitamin B12 stores [2,3]. Several observational studies have demonstrated an association between long-term PPI use and reduced vitamin B12 levels, particularly in patients on high-dose or prolonged therapy [2,3]. In contrast, folate absorption occurs predominantly in the proximal small intestine and is not dependent on gastric acidity, making a direct causal relationship between PPI use and folate deficiency less well established [5].

The concomitant folate deficiency warrants separate consideration because its relationship with PPI therapy is considerably less established than that of vitamin B12 deficiency. Folate is required for nucleotide synthesis and normal cellular replication, and deficiency contributes to defective DNA synthesis and megaloblastic erythropoiesis [9]. Folate deficiency may result from inadequate dietary intake, alcohol use, malabsorption, medication effects, or increased requirements associated with increased cellular turnover [9]. In both patients, serum folate was markedly reduced (1.0 µg/L). Neither reported dietary restriction nor alcohol misuse, and celiac serology was negative; Patient 2 also had a normal fecal elastase. Therefore, no definite cause for the folate deficiency was established. Increased folate utilization in the setting of severe ineffective erythropoiesis may have contributed, although this cannot be demonstrated from the available data. The coexistence of folate and vitamin B12 deficiency may therefore have contributed to the severity of impaired DNA synthesis and the resulting megaloblastic hematological abnormalities.

Although the association between PPI therapy and vitamin B12 deficiency is well recognized, severe presentations with features suggestive of hemolytic anemia remain underreported [4,6,8]. Most cases of megaloblastic anemia are identified earlier in the disease course; however, delayed recognition can lead to profound hematological derangement, as demonstrated in our patients. Importantly, these cases illustrate that such presentations can occur in relatively younger individuals, further increasing the potential for diagnostic uncertainty.

From a clinical perspective, several key lessons emerge. First, clinicians should maintain a high index of suspicion for vitamin B12 deficiency in patients receiving long-term PPI therapy, particularly when presenting with unexplained anemia [2,3]. Second, the combination of markedly elevated LDH, low haptoglobin, and a low reticulocyte count should prompt consideration of ineffective erythropoiesis [4,6], and folate levels should also be assessed [5]. Early recognition of this pattern may prevent unnecessary investigations for hemolytic disorders, particularly invasive diagnostic procedures. Finally, identification of combined deficiencies should lead to a comprehensive assessment, including medication review, dietary history, and evaluation for additional contributing factors.

This report has several limitations inherent to its retrospective nature. Original peripheral blood films were no longer available for imaging despite attempts to retrieve them from the hematology department. Total and indirect bilirubin measurements were unavailable for both patients, and serial LDH, haptoglobin, and reticulocyte measurements were not obtained during follow-up. Therefore, complete biochemical characterization at presentation and biochemical resolution of the hemolysis-like pattern during follow-up could not be demonstrated, although substantial hematological recovery was documented on follow-up full blood counts. In addition, the observational nature of these cases does not establish a causal relationship between PPI exposure and the vitamin deficiencies, particularly folate deficiency.

## Conclusions

These two cases demonstrate an unusual presentation of profound combined vitamin B12 and folate deficiency characterized by severe macrocytic anemia, markedly elevated LDH, reduced haptoglobin, and an inappropriately low reticulocyte response, creating an initial biochemical impression of hemolysis. Recognition of the discordance between hemolytic markers and the low reticulocyte count, together with the megaloblastic blood-film findings, was important in identifying ineffective erythropoiesis. Both patients showed substantial hematological recovery following vitamin replacement. In patients presenting with apparent hemolysis and macrocytic anemia, early measurement of vitamin B12 and folate and careful interpretation of the reticulocyte response may help distinguish megaloblastic anemia from peripheral hemolytic disorders and avoid unnecessary investigations. Medication review should form part of the assessment, with long-term PPI exposure considered as a potential contributing factor to vitamin B12 deficiency while alternative causes are investigated.
